# Regression of Cardiac Rhabdomyomas Producing a Severe Aortic Stenosis: Case Report and Discussion of the Literature

**DOI:** 10.3390/diagnostics14050470

**Published:** 2024-02-21

**Authors:** Vlasta M. E. Fesslova, Martina Evangelista, Luciane Piazza, Antonio Saracino, Andreea Andronache, Carmelina Chiarello, Alessandro Varrica, Alessandro Giamberti, Alessandro Frigiola

**Affiliations:** 1 Center of Fetal Cardiology, IRCCS Policlinico San Donato, San Donato, 20097 Milan, Italy; 2 Department of Pediatric and Adult Congenital Cardiology, IRCCS Policlinico San Donato, San Donato, 20097 Milan, Italy; martina.evangelista@grupposandonato.it (M.E.); luciane.piazza@grupposandonato.it (L.P.); antonio.saracino@grupposandonato.it (A.S.); andreeaalina.andronache@grupposandonato.it (A.A.); 3 Department of Congenital Cardiac Surgery, IRCCS Policlinico San Donato, San Donato, 20097 Milan, Italy; carmelina.chiarello@grupposandonato.it (C.C.); alessandro.varrica@grupposandonato.it (A.V.); alessandro.giamberti@grupposandonato.it (A.G.); alessandro.frigiola@grupposandonato.it (A.F.)

**Keywords:** rhabdomyoma, fetus, fetal echocardiography, aortic stenosis

## Abstract

We describe a case of a voluminous rhabdomyoma (R) detected by fetal echocardiography at 32 weeks’ gestation (w.g.) obstructing the left ventricular inflow and aortic outflow tract, with a moderate aortic gradient at birth, not needing immediate surgery. At follow-up, the mass progressively regressed, leaving the aortic valve partly damaged, with a gradient that increased to a maximum of 100 mmHg at 9 years. The girl was then operated on successfully by a plasty of the aortic valve. The literature regarding R is discussed.

## 1. Introduction

Rhabdomyomas (R) are the most common cardiac tumors detectable in fetuses. Cardiac tumors are rare, accounting for approximately 1% of all cardiac anomalies. R are evident at ultrasound as highly echogenic masses, at times multiple, originating from the cardiac walls or from the interventricular septum [1,2]. The outcome of affected cases depends on their hemodynamical impact—a potential obstruction of cardiac inflow or outflow tracts [3,4], problems of cardiac rhythm, or tachyarrhythmia, at times complicated by fetal hydrops that may be an initial referral for fetal echocardiography [5]. Also, there is a known possibility of association with tuberous sclerosis (TS), reported in a discrete number of cases and frequently associated with TSC1 and TSC2 gene anomalies [6,7,8,9,10]. The number and size of cardiac R are variable; they can occur in the late first or the second trimester and the growth of the masses in utero is also variable—from the beginning of the second trimester onward, usually until the mid-third trimester [2,11]. The hemodynamic impact of the masses determines a potential need of postnatal surgery. Once the baby is born without problems, the cardiac masses of R tend gradually to regress spontaneously [12,13]. Recently, a favorable experience with the faster regression of R was reported with therapy using the mTOR inhibitor everolimus/sirolimus [14,15,16,17,18].

## 2. Case Report

**Figure 1:**
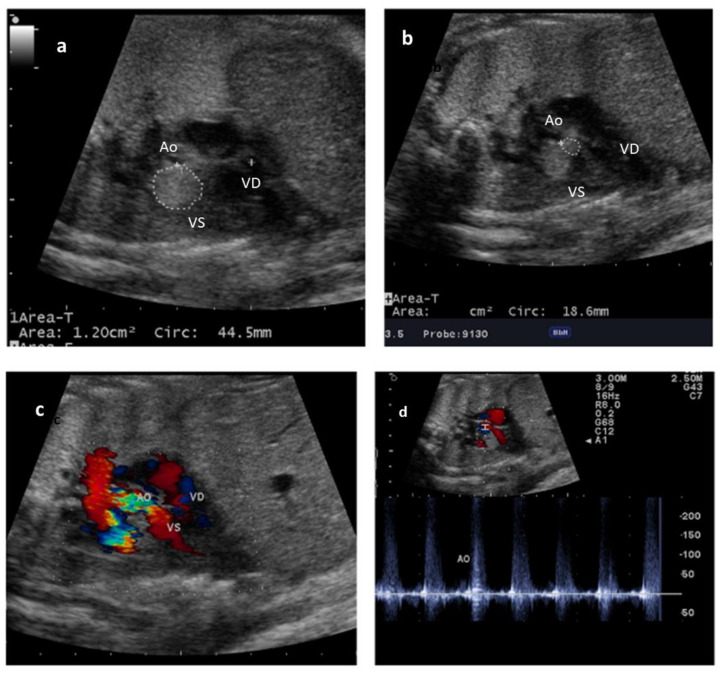
Fetal echocardiography at 32 w.g. A voluminous mass is evident in the left ventricle (VS) close to the inflow (**a**) and a smaller mass in the outflow toward the aorta (Ao) in the right panel (**b**). Color Doppler image is seen in panel (**c**) with a turbulent flow in aorta and pulsed Doppler of aorta in panel (**d**) with a max gradient of 200 cm/s at pulsed Doppler. VD—right ventricle.

A 30-year-old healthy woman in her second pregnancy, after a previous normal pregnancy with a healthy child, was sent at 32 w.g. to our center following a finding of a mass in the heart after an obstetrical scan. No familiar history of congenital heart defects or relevant anomalies were reported. The patient did not take drugs during this pregnancy; she had no gestational diabetes and no other fetal organ problems were found during routine gynecological screening. A normal obstetrical scan was reported at 20 w.g. and nothing was noted in the subsequent scans during the second trimester until the recent suspicion at 32 w.g. The fetal echocardiography in our center showed normal principal cardiac anatomy: situs solitus, normal atrioventricular and ventriculoarterial connections, two proportionate ventricles, and normal great arteries. Within the left ventricle (LV), a voluminous echodense mass (area 1.20 cm^2^, circumference 44.5 mm) was found in the inlet portion, close to the mitral valve and a smaller mass (circumference 18.6 mm) were found protruding into the aortic outflow, leaving only a small free space around the valve with an accelerated aortic flow, with pulsed Doppler velocity at upper limits (Figure 1).

The mitral valve presented only a reduced flow in Doppler imaging. No other suspicious nodes were visualized. The left ventricular (LV) contractility was normal (shortening fraction: 30%). Diagnosis of multiple R was performed, without abnormal findings in other fetal organs. The decision whether to perform a CNS MRI was postponed. Fetal MRI was not considered, prospecting to perform it after birth. We followed up the case until 36 w.g. when a cesarean section was performed, fearing further hemodynamic impact of the mass, mainly to the aortic flow.

**Figure 2:**
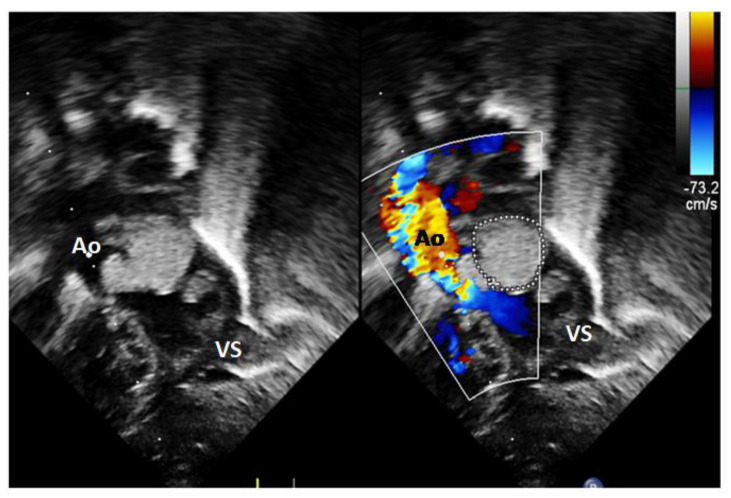
Echocardiography at birth: both echodense masses are seen, with a turbulent aortic flow. Two masses are evident in the left ventricle (LV), a bigger one in the inflow, surrounded by a circle, and a smaller one in the aortic outflow tract (x). VS—left ventricle, Ao—aorta.

A baby girl was born, in good general conditions, birth weight: 3.150 kg. No other extracardiac problems were found. Apgar 8–9. The appearance of the mass in the LV inflow was unchanged, the mitral valve presented a good diastolic flow and a mild regurgitation, while a smaller mass could be seen in the aortic area obstructing the aortic flow—with a maximum systolic gradient reaching 50 mmHg at continuous wave (CW) Doppler (see Figure 2 and the video in the Supplementary Materials).

In the following days, the aortic gradient remained at 42–50 mmHg. The LV contractility was normal. There were no rhythm problems, and the ECG was normal. Neurological evaluation with EEG was normal, and CNS MRI did not show specific lesions, so the TS could be excluded; abdominal echography and dermatological examination were also normal. The baby was discharged; no surgery was considered necessary. At follow-up, the aortic gradient remained at 40 mmHg. No epileptic crisis occurred and EEG remained normal. A CNS MRI repeated at 18 months and genetic testing for TS were negative.

At echocardiographic follow-up, conducted at 6–8 months intervals, the large cardiac mass progressively regressed spontaneously as well as the smaller aortic mass, leaving the maximum aortic gradient in systole at CW Doppler around 48–57 mmHg, medium 28 mmHg, with a mild aortic insufficiency, with an apparently damaged thickened aortic valve. Mitral insufficiency disappeared. No rhythm problems were revealed at repeated ECG Holter recordings.

**Figure 3:**
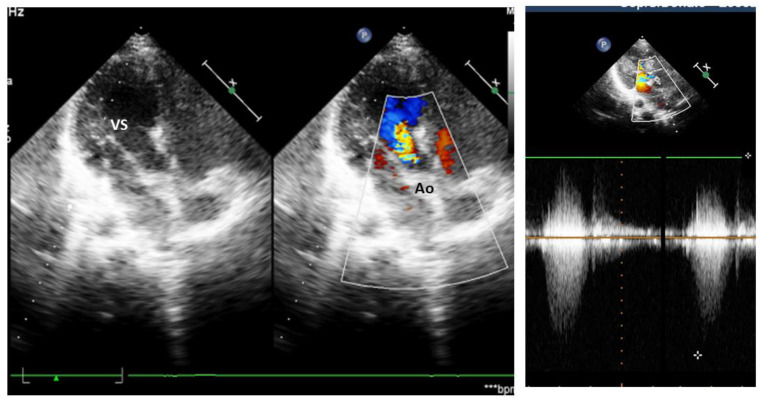
Echocardiography at 9 yrs. Only a small echodensity of a residual tumor is evident in the interventricular septum, with turbulent flow toward aorta, evident at color Doppler and with increased velocity at continuous wave Doppler at the right panel. (VS—left ventricle, ao—aorta).

At 9 years, the large LV mass almost disappeared, leaving a minimal echodensity in the superior interventricular septum, and around the aortic valve, there were only remnants of the mass, but the valve looked more severely damaged, with an abnormal opening and non-coaptation and the maximum aortic gradient increased, reaching 88–100 mmHg at CW Doppler (Figure 3).

Therefore, it was decided to plan a surgical correction, and the probability of a Ross operation was taken into consideration. The child was, however, asymptomatic, reasonably grown up.

In the operating theatre, it was possible to perform a plasty of the aortic valve with a resection of a fibrous circumferential rim, instead of a Ross operation.

The girl was discharged after an uneventful postoperative period, clinically well, showing at echocardiography a trivial aortic regurgitation and a good valve opening with a residual mild systolic gradient (of 20 mmHg at CW Doppler) with good LV function. She is now 12 years old, well, and with unchanged features at echocardiography.

## 3. Discussion

Rhabdomyomas are easy to diagnose in utero, due to their echodensity and intramural localization. They are common tumors found in fetal life, and they need to be distinguished from fibromas—which are also intramural but more rare. R can be single, but they are often multiple with more masses within the heart. Their growth in utero is variable; sometimes, these tumors can be found of a discrete dimension already in the early second trimester; however, during follow-up, other masses can be detected in the cardiac walls, in the papillary muscles and, rarely, also in atria, as a multiple rhabomyomatosis. Rare localization of R in the pericardium was described and also its familial occurrence [2,16]. Histologically, R are characterized by “spider cells”.

Association with other cardiac, extracardiac, or chromosomal anomalies is rare; of the cardiac anomalies, hypoplastic left heart, tetralogy of Fallot, and endocardial fibroelastosis have been described, and of the extracardiac anomalies, polycystic kidneys and cleft palate and clubfoot have been reported. Coexistence of cardiac rhabdomyoma with trisomy 21 has been reported in a few children and adults, but only one trisomy 21 case was detected prenatally. Other chromosomal anomalies were documented in the infancy period, including one case of trisomy 13 and one of trisomy 18, both with a single ventricular rhabdomyoma without TS [19,20,21].

Prenatal counselling [22] regards two aspects: the first one—a possible hemodynamic obstruction in large tumors or arrhythmias that can occur in fetal life and lead to fetal hydrops; and the second one—very important—a possible association with TS with specific findings on genes TSC1 and TSC2 [6,7,8,9,10]. Classically, TS is associated with cases with multiple masses (rhabdomyomatosis). TS is an autosomal dominant genetic disease involving multiple organs, including the skin, central nervous system, heart, eyes, kidneys, and lungs; polycystic kidneys may be associated with Bourneville syndrome [9]. Also, the genetic heterogenicity of TS is known, with possible associations with different loci [23]. Presence of TS can be detected by magnetic resonance imaging (MRI) in the later period in utero or after birth with findings of subependymal or subcortical tubers [8]. Frequency of association of R to TS is variable in different series, around 51–86% [6]. The CNS noduli of TS often progress after birth; effectively almost all cases with TS suffer from epileptical crisis needing specific treatment and, rarely, an evolution to CNS astroglioma may occur, often requiring a neurosurgical extirpation [10].

From the large series and a meta-analysis, it is evident that there are mostly multiple R to determine outcomes both in utero and after birth [19,20]. Sometimes, heart failure may occur already in utero, both due to obstruction and /or tachyarrhythmias, as mentioned above. Also, rhythm problems may persist after birth and postnatal evidence of signs of the preexcitation syndrome may sometimes be found [24]. Indication for surgery is usually given soon after birth [4] in large obstructing masses, but one case of a delayed surgery was reported in the literature with a necessity of excision of a subaortic ridge residual after a spontaneous regression of the tumor [3]. Effectively, this case may be put in comparison to our case.

Generally, the growth of R masses interrupts after birth and they spontaneously regress postnatally in cases associated with TS and those not associated with TS [12,13]. Spontaneous regression of the masses is explained by a mechanism of apoptosis (programmed cells death), in absence of inflammation, as f.ex occurs in myofibromatosis [25,26,27]. During the last decade, a favorable experience with the quicker regression of R has been reported with a therapy using the mTOR inhibitor everolimus/ sirolimus [14,15,16,17,18]. This treatment is useful mainly in large voluminous masses that can cause obstruction. Also, some cases were treated with this therapy in utero, with good results [18]. Our case presents some peculiarities: despite a double presence of cardiac masses that could have been associated with TS, neurological examinations and genetic tests excluded its presence. Moreover, the case is interesting regarding the presence of aortic outflow obstruction since the third trimester of gestation, but with an aortic gradient that remained stable, acceptable, and without a clear postnatal necessity to intervene immediately, and the aortic gradient remained almost identical for a long period postnatally. During these years, the large mass in the LV regressed almost completely and the smaller mass around the aortic valve also became smaller, but the valve was progressively more damaged, with a worsened opening of the valve cusps and with an increased systolic gradient. So, at 9 years, surgery was indicated and a valve plasty was successfully performed. An increase in the aortic gradient can effectively occur also in native aortic valve stenosis, the valve often being dysplastic [28,29]. In our case, the worsening of the lesion and of the gradient was due to the retraction due to regression of the tumor mass. The treatment of the native aortic valve stenosis depends not only on the gradient but also on the morphological aspect of the valve. When the valve is not particularly dysplastic, the option in almost all centers is for percutaneous valvulotomy; otherwise, surgery is considered—a plasty of the valve, replacement of the valve, or a Ross Konno procedure is indicated.

## 4. Conclusions

Our case shows a spontaneous regression of R that is known, but its particularity is in its evolution toward severe damage of the aortic valve at follow-up. To the best of our knowledge, only the case reported in the series of Smythe et al. [3] could be compared to our case. No other similar cases were reported in the literature or found in a meta-analysis study [19].

## Data Availability

Data are available from the corresponding author upon reasonable request.

## Supplementary Materials

The following supporting information can be downloaded at: https://www.mdpi.com/article/10.3390/diagnostics14050470/s1, Video clip of echocardiography at birth showing the echodense mass in the left ventricle and aortic outflow, with turbulent aortic flow.
